# Making the invisible visible: using national surveillance data to identify people experiencing homelessness in England with COVID-19

**DOI:** 10.1017/S095026882300033X

**Published:** 2023-02-28

**Authors:** Fernando Capelastegui, Joe Flannagan, Elizabeth Augarde, Elise Tessier, Dimple Chudasama, Gavin Dabrera, Theresa Lamagni, Ines Campos-Matos

**Affiliations:** 1 National COVID-19 Epidemiology Cell, UK Health Security Agency, 61 Colindale Ave, London, NW9 5EQ, UK; 2 Department of Health and Social Care, Office of Health Improvement and Disparities, 39 Victoria Street, London, SW1H 0EU, UK

**Keywords:** COVID-19, England, homelessness, SARS-CoV-2

## Abstract

Persons experiencing homelessness (PEH) or rough sleeping are a vulnerable population, likely to be disproportionately affected by the coronavirus disease 2019 (COVID-19) pandemic. The impact of COVID-19 infection on this population is yet to be fully described in England. We present a novel method to identify COVID-19 cases in this population and describe its findings. A phenotype was developed and validated to identify PEH or rough sleeping in a national surveillance system. Confirmed COVID-19 cases in England from March 2020 to March 2022 were address-matched to known homelessness accommodations and shelters. Further cases were identified using address-based indicators, such as NHS pseudo postcodes. In total, 1835 cases were identified by the phenotype. Most were <39 years of age (66.8%) and male (62.8%). The proportion of cases was highest in London (29.8%). The proportion of cases of a minority ethnic background and deaths were disproportionality greater in this population, compared to all COVID-19 cases in England. This methodology provides an approach to track the impact of COVID-19 on a subset of this population and will be relevant to policy making. Future surveillance systems and studies may benefit from this approach to further investigate the impact of COVID-19 and other diseases on select populations.

## Introduction

Persons experiencing homelessness (PEH) or rough sleeping are likely to be disproportionately impacted by infectious diseases such as coronavirus disease 2019 (COVID-19) due to poor underlying health, undiagnosed health conditions increasing the risk of complications, limited access to sanitation and vaccinations and the sometimes crowded conditions of shared accommodation [1]. The homeless charity crisis estimates that the ‘core’ number of people experiencing some form of homelessness in England was approximately 200 000 in 2021 [2]. In England the highest concentration of rough sleeping and homelessness is in London. The burden of homelessness is also high in the North West, South East and South West with the demographic of PEH heavily weighted towards male individuals 18–64 years of age [3–5].

Routine surveillance of health outcomes in PEH is challenging, in part because surveillance records rarely contain information about individual's housing status and may not be kept up to date [6]. The impact of COVID-19 on PEH in England is yet to be fully described, despite policy measures that were put in place in March 2020 at the early stages of the pandemic, such as the ‘Everyone In’ campaign that provided temporary accommodation to PEH [7]. Currently, there is no national dataset of COVID-19 cases in PEH; consequently, this report outlines a novel surveillance method that was developed to address the lack of data on this population and uses the data generated by this method to illustrate some epidemiologic differences between COVID-19 infection in this population compared to the general population.

## Methods

To identify a case in the UKHSA national COVID-19 dataset who was likely to be experiencing homelessness or rough sleeping a ‘case phenotype’ was developed by seeking and incorporating the feedback of experts in the field.

A reference list of addresses designated to providing temporary accommodation and shelter to PEH was collated by the Office of Health Improvement and Disparities. The list was compiled using data provided by NHS England, the Greater London Authority, Homeless Link and Housing Justice. The data included addresses permanently designated for accommodation for PEH, such as hostels, as well as addresses temporarily used for this purpose, such as night shelters and hotels procured for accommodation under the ‘Everyone In’ campaign. For some addresses, specific information was available, such as size, target demographic and active dates.

In a process originally developed during the COVID-19 pandemic to identify vulnerable cases, such as care home residents and those in prisons [8], UKHSA routinely matches residential address information (reported at time of test) of test-confirmed COVID-19 cases in England to a national address database. This allows cases to be linked to a Unique Property Reference Number (UPRN) and Basic Land and Property Unit (BLPU), which provides the specific location and property use respectively [8]. The reference list of addresses was matched using this same process.

The UKHSA National COVID-19 case dataset was queried, and cases were linked to the reference list of addresses using UPRN. Data were included from 24 March 2020 when the UK government announced the first ‘lockdown’ in England to 07 March 2022.

Following linkage, the criterion for inclusion were:

- Any test-confirmed COVID-19 case, where the residential address matches to a known homeless shelter or accommodation using UPRN.

OR

- Any case with ‘nfa’ or ‘no fixed abode’ in their address field

OR

- Any case with an NHS pseudo postcode of ‘ZZ993VZ’ which are supplied by The Office for National Statistics (ONS) and used in the NHS to denote in patient records when a case has no fixed abode.

Once this cohort was identified, the following cases were excluded through manual data cleaning:

- Any case with a specimen date that did not coincide with the time frame that the accommodation they matched to was active (if known), for example certain temporary accommodations such as hotels used for the ‘Everyone In’ campaign which were reopened to the public following the easing of national restrictions.
- Cases with addresses associated with a prison BLPU code (CC03, CC03HD, CC03PR) or any case with address information related to a foreign address, who can sometimes be assigned NHS pseudo postcodes.
- Cases where patient sex was unknown.

The study cohort was enhanced with data UKHSA holds on COVID-19 cases, including ethnicity and mortality. Ethnicity is derived from test information, and where missing or conflicting from Hospital Episode Statistics database. Further details on surveillance data that UKHSA holds are described comprehensively elsewhere [9, 10]. For this analysis, mortality was defined using the 60-day definition as outlined by UKHSA;

- ‘A death in a person with a positive severe acute respiratory syndrome coronavirus 2 (SARS-CoV-2) test and either died within 60 days of the first specimen date of the most recent infection or died more than 60 days after the first specimen date of the most recent infection, only if COVID-19 is mentioned on the death certificate’[10].

A comparison between the cases identified as PEH and all cases in England for the specified time period was carried out to highlight demographic differences between these populations.

## Results

A total of 1835 cases were identified as PEH. Of these 1649 cases were identified via address matching. A further 127 were identified as having no fixed abode via an NHS pseudo ZZ postcode, and 39 were identified as having ‘no fixed abode’ or ‘nfa’ in their address. A total of 237 records did not have an NHS number (12.9%). Cases matched to a total of 397 unique reference address UPRNs. The greatest number of cases identified at a given UPRN over the study period was 40, with a median of 7.

Approximately a third (29.8%) of PEH cases we identified had a residential address based in London, followed by the North West (14.4%).

There were 1152 males (62.8%) and 683 females (37.2%) identified; the median age was 35 (IQR = 23) and 23 (IQR = 16) years old respectively. There were 77 cases under the age of 18. Most cases identified were of White ethnicity (63.8%), followed by Black ethnicity (12.6%). Ethnicity was unknown for 203 cases (11.1%).

There were 25 deaths within 60 days of earliest specimen date (1.4%) in PEH during the study period; 19 in males and 6 in females.

**Fig. 1.**
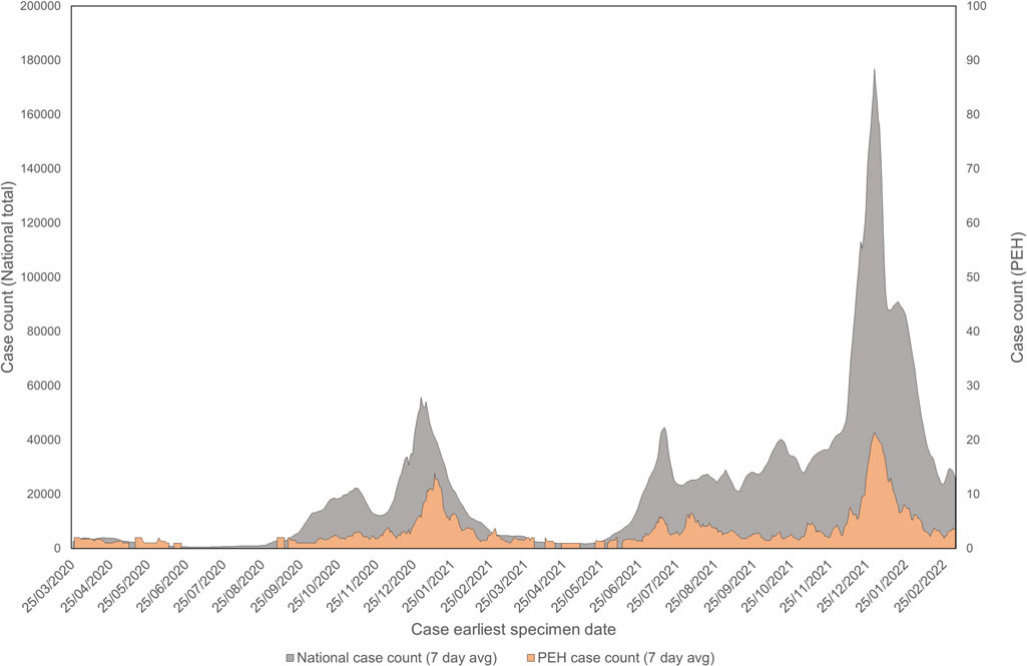
Case counts (7-day average) of PEH over national case counts (7-day average) in England between 24 March 2020 and 07 March 2022 by earliest specimen date.

The distribution of PEH cases over time displays correlation with the national case trend at various stages of the pandemic (Fig. 1) and indicated a greater proportion of cases were PEH at the start of the pandemic. Complete data can be found in Table 1 and Appendix Figure S1.

**Table 1.** Descriptive characteristics of PEH alongside total national case data for England from 24 March 2020 to 07 March 2022

	PEH			All COVID-19 cases in England		
	Female *n* (% of row)	Male n (% of row)	Total *n* (% of total)	Female *n* (% of row)	Male *n* (% of row)	Total *n* (% of total)
All	683 (37.2)	1152 (62.8)	1835 (100)	8 654 323 (53.5)	7 516 634 (46.5)	16 170 957 (100)
Age (Avg)	29.4	37.2	34.2	35.4	34.7	35.1
Case identification method						
UPRN match	645 (39.1)	1004 (60.9)	1649 (89.9)	NA	NA	NA
NHS pseudo postcode	29 (22.8)	98 (77.2)	127 (6.9)	NA	NA	NA
‘nfa’ or ‘no fixed abode’ in address	11 (28.2)	28 (71.8)	39 (2.1)	NA	NA	NA
Age group						
0–19	194 (62)	119 (38)	313 (17.1)	2 115 337 (50.8)	2 045 908 (49.2)	4 161 245 (25.7)
20–29	248 (44.8)	306 (55.2)	554 (30.2)	1 545 862 (54.8)	1 272 867 (45.2)	2 818 729 (17.4)
30–39	104 (29)	255 (71)	359 (19.6)	1 571 717 (55.6)	1 257 466 (44.4)	2 829 183 (17.5)
40–49	65 (21.5)	238 (78.5)	303 (16.5)	1 355 242 (55.2)	1 098 714 (44.8)	2 453 956 (15.2)
50–59	41 (22)	145 (78)	186 (10.1)	1 043 679 (53.6)	903 571 (46.4)	1 947 250 (12)
60–69	10 (15.6)	54 (84.4)	64 (3.5)	514 703 (50.9)	497 312 (49.1)	1 012 015 (6.3)
70 +	21 (38.9)	33 (61.1)	54 (2.9)	502 681 (53.6)	434 695 (46.4)	937 376 (5.8)
Unknown	0 (0)	2 (100)	2 (0.1)	5102 (45.5)	6101 (54.5)	11 203 (0.1)
Region						
East Midlands	30 (34.5)	57 (65.5)	87 (4.7)	744 151 (53.6)	645 422 (46.4)	1 389 573 (8.6)
East of England	112 (50.9)	108 (49.1)	220 (12)	969 848 (53.3)	850 829 (46.7)	1 820 677 (11.3)
London	208 (38.1)	338 (61.9)	546 (29.8)	1 373 261 (53.4)	1 199 310 (46.6)	2 572 571 (15.9)
North East	13 (24.1)	41 (75.9)	54 (2.9)	451 505 (53.9)	385 846 (46.1)	837 351 (5.2)
North West	77 (29.2)	187 (70.8)	264 (14.4)	1 231 698 (53.8)	1 058 863 (46.2)	2 290 561 (14.2)
South East	57 (44.2)	72 (55.8)	129 (7)	1 311 890 (53.3)	1 149 554 (46.7)	2 461 444 (15.2)
South West	64 (43.2)	84 (56.8)	148 (8.1)	763 052 (53.5)	663 060 (46.5)	1 426 112 (8.8)
West Midlands	56 (40.3)	83 (59.7)	139 (7.6)	904 452 (53.6)	783 776 (46.4)	1 688 228 (10.4)
Yorkshire and Humber	37 (30.8)	83 (69.2)	120 (6.5)	856 471 (53.9)	731 282 (46.1)	1 587 753 (9.8)
Unknown	29 (22.7)	99 (77.3)	128 (7)	47 995 (49.6)	48 692 (50.4)	96 687 (0.6)
Mortality all						
60 day measure	6 (24)	19 (76)	25 (1.4)	62 927 (44.6)	78 101 (55.4)	141 028 (0.9)
Ethnicity						
Asian	36 (35.3)	66 (64.7)	102 (5.6)	774 561 (51.9)	717 353 (48.1)	1 491 914 (9.2)
Black	100 (43.1)	132 (56.9)	232 (12.6)	310 776 (55)	253 934 (45)	564 710 (3.5)
Mixed	41 (53.2)	36 (46.8)	77 (4.2)	229 366 (54.5)	191 654 (45.5)	421 020 (2.6)
Other	9 (18)	41 (82)	50 (2.7)	112 695 (51.5)	106 143 (48.5)	218 838 (1.4)
Unknown	62 (30.5)	141 (69.5)	203 (11.1)	470 881 (51.4)	444 468 (48.6)	915 349 (5.7)
White	435 (37.1)	736 (62.9)	1171 (63.8)	6 756 044 (53.8)	5 803 082 (46.2)	12 559 126 (77.7)

Does not include cases with unknown sex.

## Discussion

Being a vulnerable population, it is important to highlight the impact of COVID-19 on PEH in England to inform policy responses; however, studies using national data are currently lacking.

This study has identified a cohort of 1835 COVID-19 cases who were likely to be PEH between March 2020 and March 2022. We report that most PEH cases were young males, and that the number of cases in PEH was greatest in London. We also find that the majority of PEH cases were of White ethnicity. Our findings broadly align with the expected demographic characteristics of PEH cases which has been described in England [3, 4]. This method provides a unique, population phenotype which we can use to investigate the impact of COVID-19 on PEH.

We identified most cases in London (29.8%) and the North West (14.4%) and the fewest in the North East (2.9%). Relative to national case data, we identified a greater proportion of PEH cases in London (29.8% *vs*. 15.9%). The opposite was seen in other regions; for example the East Midlands (4.7% *vs*. 8.6%) and the South East (7% *vs*. 15.2%). It is unclear whether transmission of COVID-19 among PEH was greater in London, though it was expected as the number of PEH is highest in London as reported by the homeless charity Shelter and ONS [2, 4, 5] and consequently there are more temporary accommodations and shelters in London for PEH. These findings present counts and not rates of COVID-19 in PEH which may also help explain this.

When further comparing demographic characteristics of PEH to the national COVID-19 case data in England, there are some key differences concerning to the relative impact COVID-19 has had on PEH. Although the majority of PEH cases were of White ethnicity (63.8%), the proportion of PEH that are of an ethnic minority background was generally higher in PEH than in the national case data. For example, the proportion of cases of Black ethnicity in PEH was suggestively higher compared to the national case data (12.6% *vs*. 3.5%). This may reflect a higher risk of COVID-19 infection in PEH of Black ethnic groups, however this cannot be concluded with certainty without PEH denominators overall and by ethnicity.

Additionally, the proportion of deaths, based on an unadjusted comparison, in PEH (1.4%) *vs.* the national proportion of deaths in COVID-19 cases (0.9%) suggests poorer COVID-19 health outcomes in PEH which is an area of potential concern. Further analysis and consideration would be needed to understand these findings in more details based on differences in testing, cohort sizes as well as differences in vaccination status, sex and co-morbidities which were outside the scope of the development of these methods. Due to deductive disclosure via small numbers it was not possible to display mortality by age-bands.

These findings provide important context to public health policy measures [7] that were put in place in England to minimise the impact of COVID-19 on PEH and this methodology may help in evaluating those measures, particularly in the early stages of the planning when the crude proportion of PEH cases was higher (Appendix Fig. S1). A centralised database of shelters and accommodations would be beneficial for future surveillance of the impact of infectious diseases on this population.

A more in-depth assessment of outbreaks within shared accommodation settings and shelters is needed to provide specific details on the risk of COVID-19 transmission. Further studies could use this methodology to investigate the severity of COVID-19 in PEH through linkage to hospital records, as well as the uptake of vaccines which will be key to policy and inclusion health outcomes in this area. Similar methodologies could also be applied to other infectious diseases that disproportionately affect PEH such as Hepatitis, HIV and other respiratory diseases to identify cases based on address information.

## Limitations

It is not possible to assess the scale of the potential limitations associated with this methodology, as there is no relevant national reference data set for comparisons to be made.

These findings will likely be an underestimate of COVID-19 in PEH, primarily due to the low sensitivity of the methods to identify broader homelessness situations, and should patient address information be out of date in laboratory and electronic medical records. We attempted to address this limitation by using NHS pseudo postcodes, which are used in patient records when a case has no fixed abode, although its completeness in COVID-19 testing and hospital records is unknown.

PEH face challenges in accessing care, which affects the quality and timeliness of health records [6] and it is probable that a proportion of PEH cases in the UKHSA COVID-19 national dataset will not have had sufficient address data to meet the criteria for inclusion. Furthermore, accommodation sites are constantly being updated so some data in the reference list may be out of date or incomplete. The reference list also does not contain addresses which are confidential.

## Conclusions

This study presents evidence that PEH have been impacted by the COVID-19 pandemic. The methodology and phenotype for this population has been shown to be practical to implement and demonstrates that it could be applied to further surveillance and research on COVID-19 in PEH, and potentially also to other diseases.

## Data Availability

No data are available. This work is carried out under Regulation 3 of The Health Service (Control of Patient Information) (Secretary of State for Health, 2002) (3) using patient identification information without individual patient consent. Data cannot be made publicly available for ethical and legal reasons, that is, public availability would compromise patient confidentiality as data tables list single counts of individuals rather than aggregated data.

## Supplementary material

For supplementary material accompanying this paper visit https://doi.org/10.1017/S095026882300033X. click here to view supplementary material

## Statements and declarations

The authors declare that no funds, grants or other support were received during the preparation of this manuscript.
