# Supplementary material for: Making the invisible visible: using national surveillance data to identify people experiencing homelessness in England with COVID-19
Source: Epidemiol Infect. 2023 Feb 28;151:e51. doi: 10.1017/S095026882300033X (PMC10063863; doi:10.1017/S095026882300033X)
Supplement: Supplementary file 1 [file S095026882300033Xsup001.docx]

**Appendix to ‘Methods to identify people experiencing homelessness in England with COVID-19 using national surveillance data’**

Figure 1. Proportion of PEH cases vs national case counts (7 day average) over time in England between 24 March 2020 to 07 March 2022 by earliest specimen date
